# Transcranial Alternating Current Stimulation With the Theta-Band Portion of the Temporally-Aligned Speech Envelope Improves Speech-in-Noise Comprehension

**DOI:** 10.3389/fnhum.2020.00187

**Published:** 2020-05-29

**Authors:** Mahmoud Keshavarzi, Tobias Reichenbach

**Affiliations:** Department of Bioengineering, Centre for Neurotechnology, Imperial College London, South Kensington Campus, London, United Kingdom

**Keywords:** neural entrainment, theta frequency band, transcranial current stimulation, speech envelope, speech comprehension, speech-shaped-noise, normal hearing

## Abstract

Transcranial alternating current stimulation with the speech envelope can modulate the comprehension of speech in noise. The modulation stems from the theta- but not the delta-band portion of the speech envelope, and likely reflects the entrainment of neural activity in the theta frequency band, which may aid the parsing of the speech stream. The influence of the current stimulation on speech comprehension can vary with the time delay between the current waveform and the audio signal. While this effect has been investigated for current stimulation based on the entire speech envelope, it has not yet been measured when the current waveform follows the theta-band portion of the speech envelope. Here, we show that transcranial current stimulation with the speech envelope filtered in the theta frequency band improves speech comprehension as compared to a sham stimulus. The improvement occurs when there is no time delay between the current and the speech stimulus, as well as when the temporal delay is comparatively short, 90 ms. In contrast, longer delays, as well as negative delays, do not impact speech-in-noise comprehension. Moreover, we find that the improvement of speech comprehension at no or small delays of the current stimulation is consistent across participants. Our findings suggest that cortical entrainment to speech is most influenced through current stimulation that follows the speech envelope with at most a small delay. They also open a path to enhancing the perception of speech in noise, an issue that is particularly important for people with hearing impairment.

## Introduction

Understanding speech in noisy backgrounds such as in a loud pub or restaurant is a challenging task at which humans excel (Cherry, 1953; Bregman et al., 1990). It requires the segregation of a target speech stream from other sound sources as well as the further parsing and processing of the speech signal. The complexity of these tasks becomes evident when considering people with hearing impairment, for whom the neural signals carry a degraded representation of the sound and who consequently experience significant difficulty when background noise is loud (Dubno et al., 1984; Lorenzi et al., 2006; Koelewijn et al., 2012). Similarly, despite significant recent progress, automatic speech recognition often still performs poorly in noisy environments (Heymann et al., 2016; Chen et al., 2017).

One neural mechanism for understanding speech presumably involves the entrainment of cortical activity to the envelope of speech. Speech contains information at different time scales, such as the rates of words and syllables, and these rhythms appear in the speech envelope. Neural activity in the cortex tracks this rhythm (Aiken and Picton, 2008; Ding and Simon, 2012, 2014; Giraud and Poeppel, 2012). The tracking is larger for an attended than for an unattended speech signal (Ding and Simon, 2012; Horton et al., 2013; O’Sullivan et al., 2014), and can inform on speech comprehension (Di Liberto et al., 2015; Ding et al., 2016; Broderick et al., 2018; Vanthornhout et al., 2018; Etard and Reichenbach, 2019).

The cortical oscillatory activity can be modulated through transcranial alternating current stimulation (Herrmann et al., 2013; Helfrich et al., 2014). Presumably due to influencing the neural entrainment to speech, electrical stimulation with the speech envelope has accordingly been found to modulate the comprehension of speech in background noise (Riecke et al., 2018; Wilsch et al., 2018; Zoefel et al., 2018; Kadir et al., 2020). In particular, speech-in-noise comprehension has been observed to depend on the delay between the current waveform and the audio signal.

The speech envelope is a comparatively broad-band signal, encompassing mostly fluctuations in the delta frequency band (1–4 Hz) and the theta frequency band (4–8 Hz; Ghitza et al., 2012; Etard and Reichenbach, 2019). We have recently investigated the relative contributions of the delta-band and the theta-band portions of the current waveform, derived from the speech envelope, to the modulation of speech comprehension (Keshavarzi et al., 2020). We found that only the theta-band current waveform, but not the delta-band one, modulated the comprehension of speech in background noise.

We obtained these results by considering current waveforms that were temporally aligned to the speech signal but had different phase shifts. We found that the theta-band signal without phase shift yielded the highest speech comprehension, significantly better than that obtained for sham stimulation. However, we did not further investigate the role of temporal delays between the current waveform and the audio signal. Here we address this issue by considering how transcranial alternating current stimulation, in which the current waveform is obtained from the theta-band portion of the speech envelope and shifted by different lags, impacts speech comprehension.

## Materials and Methods

### Participants

Sixteen right-handed, native English speakers with normal hearing participated in the experiment (nine females, seven males, aged between 19 and 30 years, mean age 21.5 years). They had no history of hearing impairment, mental health problems or psychological or neurological disorders. All subjects gave informed consent to participate in the study. The experiment was approved by the Imperial College Research Ethics Committee.

### Experimental Setup

We used a PC with a Windows 7 operating system to generate the acoustic stimuli and the current waveforms digitally. A USB-6212 BNC device (National Instruments, Austin, TX, USA) that was connected to the PC was employed to convert both stimuli to analogue signals. The current waveform was passed to a splitter that was connected to two neurostimulation devices (NeuroConn, Germany). Both neurostimulation devices thus created current signals that were proportional, and time-aligned, to the received waveform. The acoustic stimuli were passed to a soundcard (Fireface 802, RME, Germany) that was connected to earphones (ER-2, Etymotic Research, Elk Grove Village, IL, USA).

### Acoustic Stimuli

**Figure 1:**
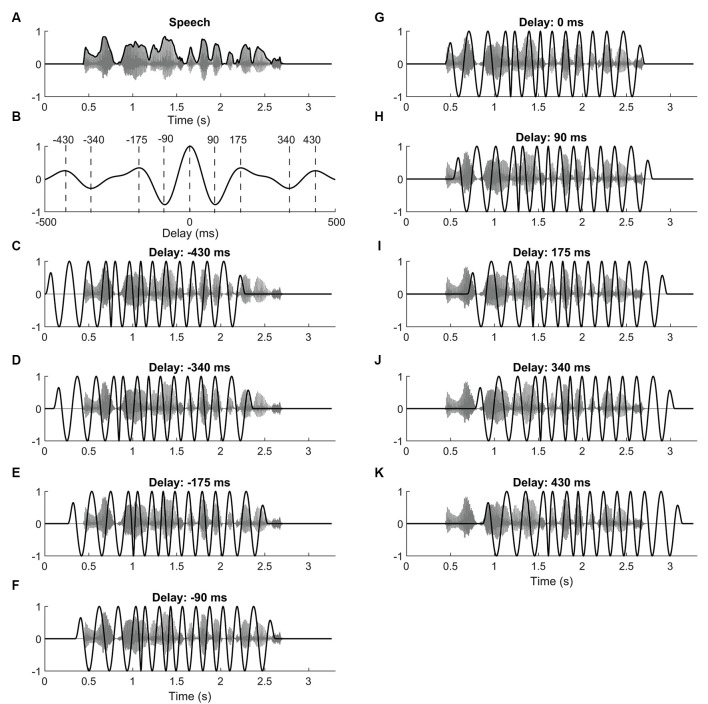
Speech stimuli and current waveforms. **(A)** Speech comprehension was determined by presenting subjects with sentences (gray) embedded in speech-shaped noise. The envelope of the sentence (black) served to define the neurostimulation waveform. **(B)** The autocorrelation of the speech envelope shows maxima at 0 ms, at ±175 ms, and at 430 ms. Minima occur at ±90 ms and at ±340 ms. **(C–K)** Subjects were simultaneously stimulated with transcranial alternating current. The current waveform (black) was derived from the theta-band portion of the speech envelope, and was shifted with respect to the speech (gray) by different delays. For the delays we chose those of the maxima and minima of the speech envelope’s autocorrelation function.

The acoustic stimuli were single, semantically unpredictable sentences corrupted by speech-shaped noise (Figure 1A). The speech-shaped-noise was spectrally matched to the speech and was created by calculating the Fourier transform of the different sentences. The phases of the different spectral components were then randomized, while the magnitude was left unchanged. The noise was then obtained by computing the inverse Fourier transform of the resulting signal.

The sentences were generated using Python’s Natural Language Toolkit (Bird et al., 2009; Beysolow, 2018). Each sentence (e.g., “A young period allows the verbal potatoes.”) consisted of seven words including five keywords which were used to evaluate the participant’s comprehension score. The TextAloud software was utilized to convert sentences to audio stimuli with a male voice. The sampling rate and the intensity of the presented speech (excluding noise) were 44,100 Hz and 65 dB SPL, respectively.

### Neurostimulation Waveforms

Ten different types of neurostimulation waveforms were used in the experiment. One type of waveform was a sham stimulus that started at the beginning of the speech stimulus and lasted for 500 ms. Smooth onsets and offsets were achieved through employing ramps with a duration of 100 ms.

The remaining nine types of waveforms were derived from the envelope of the respective target sentence (Figures 1C–K). In particular, each type of waveform differed from sentence to sentence. The envelope was computed as the absolute value of the analytical representation of the speech signal, obtained through the Hilbert transform. The speech envelope was then band-pass filtered to extract the theta frequency band [zero phase IIR filter, low cut off (−3 dB) 4 Hz, high cut off (−3 dB) 8 Hz, order 6]. Because of the band-pass filtering, the resulting waveform had a mean of zero. The obtained signal was then temporally shifted by nine different lags: 0 ms, ±90 ms, ±175 ms, ±340 ms, ±430 ms. These lags were chosen to correspond to the maxima and minima in the autocorrelation of the theta-band portion of the speech envelope (Figure 1B).

This choice of temporal lags was made so that subsequent lags would lead to neurostimulation waveforms that were as either as similar or as dissimilar from the non-shifted waveform as possible, within their temporal range. In particular, time lags at which the autocorrelation was maximal corresponded to waveforms that were rather similar to the unshifted signal. Analogously, waveforms shifted by the temporal lags of the auto correlation’s minima were particularly anti-correlated to the unshifted waveform. The correlation of the neurostimulation signal shifted by other temporal lags with the unshifted waveform led to intermediate levels of correlation or anticorrelation. We focused on the temporal shifts that corresponded to the extrema since we expected the neurostimulation waveform shifted by other delays to reflect the behavior seen at these maxima and minima.

To increase the impact of the current signals on the neural entrainment, all maxima (and minima) in the waveforms were set to the maximal (and minimal) value that was encountered in the signal. This was done by computing the analytical representation of the waveform using the Hilbert transform, by subsequently setting the amplitude to unity, and by then taking the real part of the obtained function. The resulting waveform still showed the temporal variations of the speech envelope, but the maxima and minima all had the same magnitude.

### Experimental Procedure

All experimental testing took place in a sound-proof and semi-anechoic chamber. The subjects were seated and wore earphones (ER-2, Etymotic Research, USA). Two rubber electrodes were placed adjacently left and right of the location Cz, and the two other ones at the locations T7 and T8 of the International 10-20 system. One electrode placed near Cz and the one at T7 were connected to one neurostimulation device and the remaining electrodes to the other device. The electrodes at the temporal areas served as the anodes and the ones at Cz as the cathodes. All electrodes were covered by sponge pads (35 cm^2^) wetted by a 0.9% saline solution (about 5 ml per electrode). After putting them on the participant’s head, the resistance between the electrodes connected to each device was set to below 10 kΩ. The sound stimuli and the current signals were presented through software that was custom written in Python 2.7. The resulting digital signals were then converted to analogue waveforms through a USB-6212 BNC device (National Instruments, Austin, TX, USA). This setup allowed precise control of the timing of the sound signals for the current waveforms.

To measure the maximum magnitude of the stimulation current to be used for a particular participant, a sinusoidal signal with a frequency of 3 Hz and with a duration of 5 s was presented to the subject. The signal amplitude was initially 0.1 mA and was increased to a maximum of 1.5 mA in steps of 0.1 mA. The procedure was stopped when the subject felt a skin sensation, and the amplitude used in the previous step was chosen as the maximum threshold for the stimulation current for that participant.

For each participant, we then measured the sentence reception threshold (SRT) of 50% during sham stimulation. The sham stimulus was the same as the one used in the subsequent measurements. This threshold is the signal-to-noise ratio (SNR) at which speech comprehension was 50%. The SRT was estimated through an adaptive procedure (Kollmeier et al., 1988; Kaernbach, 2001). The initial SNR was randomly selected between 0 dB to −3 dB. If the subject understood three or more keywords in a sentence correctly, the SNR value was decreased by 1 dB for the subsequent sentence, otherwise, it was increased by 1 dB. The adaptive procedure was stopped after seven reversals in the SNR or after presenting 17 sentences. The procedure was conducted four times for each subject and the final SNR was calculated as the average of the last three SNR values during the last three repetitions.

The so-established SRT was then used as the SNR for determining the influence of the current stimulation on speech comprehension. To this end, we measured the subjects’ speech comprehension during concurrent transcranial current stimulation with the 10 different current waveforms. For each waveform, we presented a subject with 25 sentences corrupted by speech-shaped noise, at the SNR that corresponded to the SRT of that subject. We simultaneously applied the current stimulation. After listening to each sentence, the subject was asked to repeat what he or she understood. The response was recorded through a microphone and manually graded by the experimenter for the percentage of correctly understood words. Each subject heard every sentence only once during the experiment.

The response was graded on the five keywords for each sentence, each of which was assigned a score of 20%. The lowest score for each sentence was therefore 0, and the highest score was 100%. For example, a subject understood four key words of a sentence correctly, the score for that sentence was 80%. The speech comprehension score for each condition was then obtained by averaging across all corresponding comprehension scores (25 trials).

A total of 250 sentences was presented during the testing session that lasted for about 80 min. The type of current stimulation varied randomly from sentence to sentence and was unknown to both the subject and the experimenter (double-blind design). After every 50 sentences, the subject had a 2-min break.

To investigate the influence of between-subject variation in the effect of the neurostimulation, we determined the best delay per subject, that is, the delay that leads to the highest speech comprehension score for that participant. We then measured the delay relative to this best delay. Because of the delays that we employed corresponding to the maxima and minima of the speech envelope’s autocorrelation function, they were not multiples of a certain duration. The delays measured relative to the best delay could, therefore, differ between subjects. We dealt with this irregularity in the relative delays by binning them in bins of 100 ms duration.

We also assessed the correlation between comprehension scores obtained under different stimulation conditions across the different subjects. We thereby excluded data from an individual subject as an outlier if the corresponding comprehension score was more than a 1.5 interquartile range above the upper quartile or below the lower quartile of the population data.

## Results

**Figure 2:**
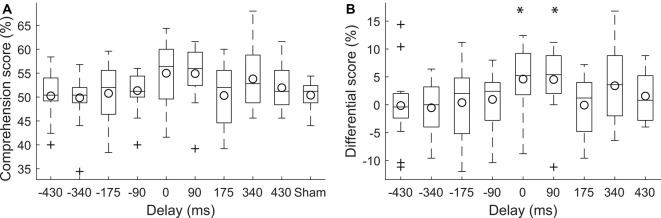
Modulation of speech comprehension by the transcranial current stimulation. **(A)** The speech comprehension at the population level for the different neurostimulation conditions is shown through box plots. The circles indicate the population means. **(B)** We carried out statistical analysis on the differences in the speech comprehension scores at the various delays and the speech comprehension score under the sham conditions. The differences at the delays of 0 ms and 90 ms were significantly larger than 0*. Stimulation at these delays accordingly led to higher speech comprehension than sham stimulation. The differential scores at the other delays did not differ significantly from zero.

We determined the speech comprehension scores of subjects while they experienced transcranial electrical stimulation with the theta-band portion of the speech envelope at different delays, as well as when they were presented with sham stimulation (Figure 2A). For the delays, we considered a range of negative and positive delays. Negative delays implied that the current waveform preceded the speech signal, whereas the current waveform lagged the audio for positive delays.

To investigate the effect of the current stimulation at the various delays on speech comprehension, we computed the difference of the corresponding speech comprehension scores and the score that was obtained during sham stimulation (Figure 2B). We found that there was statistically significant variation between the resultant differential scores (one-way ANOVA, *df* = 8, *F*: 2.12, *p* = 0.038, *η*^2^ = 0.1). *Post hoc* tests (Tukey-Kramer method) showed, however, no significant difference between the comprehension scores at the nine different delays.

We further explored whether there were delays for which the scores significantly differed from zero. We found that the comprehension scores related to the delay of 0 ms were significantly above zero (*p* = 0.03, paired two-tailed student’s *t*-test, adjusted for the nine different comparisons through the FDR correction; Benjamini and Hochberg, 1995). The comprehension scores during current stimulation at no delay were 5% ± 6% (mean and SD) higher than under sham condition. In other words, subjects understood approximately one additional keyword in four sentences, which contained 20 keywords all together. The effect size was 0.93 (Cohen’s *d* for paired samples).

The scores corresponding to delay of 90 ms were significantly larger than zero as well (*p* = 0.03, paired two-tailed student’s *t*-test, adjusted for the nine different comparisons through the FDR correction). Current stimulation at the delay of 90 ms led to subjects understanding 5% ± 6% more words than under sham stimulation, with an effect size of 0.99 (Cohen’s *d* for paired samples).

We wondered whether the variation in the speech comprehension scores across the different subjects could be explained by the individual SRT of that subject, and/or by the current stimulation level that was employed for the corresponding participant. Both the SRT and the current intensity did indeed vary across subjects: the SRT had a population average of –3.0 ± 1.3 dB (mean and SD), and the current intensity of 1.0 ± 0.3 mA (mean and SD). To investigate their influence on speech comprehension, we employed a linear regression model to predict the speech comprehension scores at 0 ms and 90 ms from these two variables. At the delay of 0 ms, we found neither a significant influence of the subject’s SRT (*p* = 0.7, FDR adjustment for two comparisons) nor of the current stimulation level (*p* = 0.7, FDR adjustment for two comparisons). Likewise, the speech comprehension scores at 90 ms were neither predicted by the SRT (*p* = 0.3, FDR adjustment for two comparisons) nor by the current magnitude (*p* = 0.4, FDR adjustment for two comparisons).

**Figure 3:**
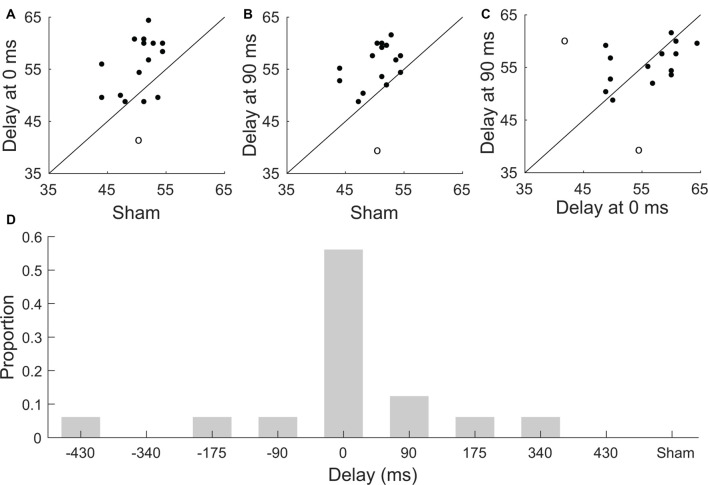
Subject-to-subject variability in speech comprehension. **(A–C)** Scatter plots of comprehension scores for individual participants. The diagonal line denotes identical scores. Outliers are indicated through circles. **(A)** Comprehension scores obtained for the sham condition vs. those of the stimulation for a delay of 0 ms. There is no significant correlation between the scores obtained for these conditions (Pearson, *p* = 0.1). **(B)** Scatter plot of comprehension scores obtained for the sham stimulation vs. those of the stimulation for a delay of 90 ms. The correlation between these conditions is not significant (Pearson, *p* = 0.1). **(C)** The correlation between the comprehension scores obtained at a delay of 0 ms and at a delay of 90 ms is not significant either (Pearson, *p* = 0.06). **(D)** Distribution of the best neurostimulation delay amongst the study participants. The majority of subjects exhibited the best speech comprehension at no delay between the neurostimulation waveform and the speech signal. The distribution of the best delays per participant differed significantly from a uniform one.

We also investigated whether subjects that scored highly when presented with current stimulation at either 0 ms or 90 ms would also exhibit high comprehension scores under sham stimulation (Figures 3A,B). However, we found no significant correlation between the speech comprehension scores at a delay of 0 ms and sham (one outlier excluded, Pearson’s correlation coefficient *r* = 0.4, *p* = 0.1), and neither was the correlation between the scores at a delay of 90 ms and those obtained under sham stimulation significant (one outlier excluded, Pearson’s correlation coefficient *r* = 0.4, *p* = 0.1). The comprehension scores obtained for stimulation at no delay and those for a delay of 90 ms were not significantly correlated either (two outliers excluded, Pearson’s correlation coefficient *r* = 0.5, *p* = 0.06, Figure 3C). However, the latter correlation coefficient approached statistical significance, suggesting that a larger pool of participants may lead to significantly correlated comprehension scores at these delays.

We further explored the inter-subject variability in the modulation of speech comprehension by the different neurostimulation types. In particular, we investigated whether the best delay, that is, the delay of the transcranial current waveform that led to the highest speech comprehension score for a particular subject differed between the study participants. We found, however, that the majority of the study participants, 57%, had the best delay of 0 ms. We determined whether the distribution of the best delays differed significantly from a uniform one through the Frosini and the Hegazy-Green tests (Hegazy and Green, 1975; Frosini, 1987). We found that the distribution was significantly non-uniform (Figure 3D; Frosini, *p* = 2e-16, *B* = 3.76; Hegazy-Green, *p* = 2e-16, *T* = 1.2).

**Figure 4:**
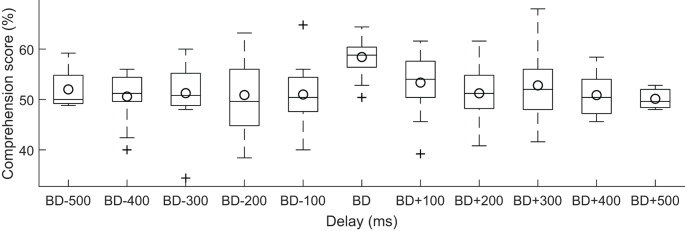
Dependence of speech comprehension on the delay of the neuro stimuliation waveform with respect to the best delay per subject. For each subject, we measured the delay of the current stimulation with respect to the best delay (BD) for that subject. We then divided the delays into bins of a duration of 100 ms each, and determined which bin that the delay fell into. The speech comprehension scores showed no significant variation with this relative delay (box plots, circles denote the population mean).

Although the best delays were relatively consistent across subjects, there was nonetheless some variation in this best delay. We wondered if the speech comprehension scores would exhibit stronger modulation by the current stimulation when the delay was measured relative to each subject’s best delay. We found, however, that this adjustment did not yield a significant dependence of the speech comprehension scores on the relative delay (Figure 4, ANOVA, *p* = 0.95).

## Discussion and Conclusion

Our study showed that current stimulation with the theta-band portion of the speech envelope benefits the comprehension of speech in noise most if it occurs at no delay, or at most at a slight delay, with respect to the audio signal. Moreover, we showed that, under this condition, the transcranial current stimulation leads to an enhancement of speech-in-noise comprehension as compared to sham stimulation. The latter result replicated our finding from an earlier study where we investigated the influence of phase shifts of the current on speech-in-noise comprehension (Keshavarzi et al., 2020). For negative delays or positive delays of 175 ms or larger, we did not find a significant difference to sham stimulation. It, therefore, appears that current stimulation at these longer delays does not affect the neural processing of the acoustic signal, neither in a beneficial nor in an inhibitory manner.

We found that the current stimulation at delays of both 0 ms and 90 ms improved speech comprehension. This finding appeared unexpected since the waveform shifted by 90 ms was anticorrelated to that without a temporal shift (Figure 1B). If neurostimulation without a temporal delay improved speech comprehension, we, therefore, expected that stimulation without a delay would lead to worse speech recognition scores. However, since both delays led to improved comprehension of speech in background noise, we conclude that the best delay is presumably in between 0 and 90 ms. Future studies may employ a finer spacing of time delays to obtain a fuller map of the influence of the temporal delay on speech comprehension and to obtain a better estimate of the optimal delay.

An important question regarding the modulation of speech-comprehension through transcranial current stimulation is the subject-to-subject variability. Some studies found that the influence of a main parameter of the stimulation—either the temporal delay or a phase shift—on speech comprehension varied considerably between subjects (Riecke et al., 2018; Wilsch et al., 2018; Zoefel et al., 2018). Adjusting this parameter relative to the one that yielded the largest effect on speech comprehension was, therefore, necessary to observe significant effects on the population level. However, other studies did not find such a significant variation between subjects (Kadir et al., 2020; Keshavarzi et al., 2020). Instead, they found that the parameter that yielded the highest speech comprehension was relatively consistent between subjects and that the modulation of speech comprehension on the population level emerged clearest when this parameter was not adjusted on an individual basis. Here we observed the latter behavior. The latency that yielded the largest improvement in speech comprehension did not vary largely between subjects. Also, the population-level effects of the current stimulation on speech comprehension emerged only when the latency was not measured relative to the best latency per subject. This indicates that the neural mechanisms for speech processing upon which the current stimulation acts are relatively consistent between subjects.

In summary, our study showed that current stimulation can not only modulate but improve the comprehension of speech in noise as compared to sham stimulation. Together with our previous study on phase changes, our current work demonstrates that this improvement happens if the current signal follows the theta-band portion of the speech envelope, when it is temporally aligned to the acoustic waveform, and when it has no additional phase shift. Future work is required to identify the neural mechanisms through which the enhancement of speech comprehension is achieved, as well as to optimize the current waveforms to potentially improve speech-in-noise comprehension yet further.

## Data Availability Statement

The datasets generated for this study are available on request to the corresponding author.

## Ethics Statement

The studies involving human participants were reviewed and approved by Imperial College Research Ethics Committee. The participants provided their written informed consent to participate in this study.

## Author Contributions

MK and TR designed the research, interpreted the data and wrote the article. MK carried out the experimental study and analyzed the data.

## Conflict of Interest

The authors declare that the research was conducted in the absence of any commercial or financial relationships that could be construed as a potential conflict of interest.
